# LncRNA PITPNA‐AS1/miR‐223‐3p/PTN axis regulates malignant progression and stemness in lung squamous cell carcinoma

**DOI:** 10.1002/jcla.24506

**Published:** 2022-05-19

**Authors:** Bi‐hao Peng, Yu‐fei Ji, Xiao‐jian Qiu

**Affiliations:** ^1^ The Second Clinical Medical School Nanchang University Nanchang China; ^2^ Department of Respiratory and Critical Care Medicine Beijing Tiantan Hospital, Capital Medical University Beijing China

**Keywords:** LUSC, miR‐223‐3p, PITPNA‐AS1, PTN, stemness

## Abstract

**Background:**

Long noncoding RNAs (lncRNAs) are a kind of molecule that cannot code proteins, and their expression is dysregulated in diversified cancers. LncRNA PITPNA‐AS1 has been shown to act as a tumor promoter in a variety of malignancies, but its function and regulatory mechanisms in lung squamous cell carcinoma (LUSC) are yet unknown.

**Methods:**

The mRNA and protein expression of genes were examined by RT‐qPCR, western blot, and IHC assay. The cell proliferation, migration, invasion, and stemness were detected through CCK‐8, colony formation, Transwell and spheroid formation assays. The CD44^+^ and CD166^+^‐positive cells were detected through flow cytometry. The binding ability among genes through luciferase reporter and RNA pull‐down assays. The tumor growth was detected through in vivo nude mice assay.

**Results:**

The lncRNA PITPNA‐AS1 had increased expression in LUSC and was linked to a poor prognosis. In LUSC, PITPNA‐AS1 also enhanced cell proliferation, migration, invasion, and stemness. This mechanistic investigation showed that PITPNA‐AS1 absorbed miR‐223‐3p and that miR‐223‐3p targeted PTN. MiR‐223‐3p inhibition or PTN overexpression might reverse the inhibitory effects of PITPNA‐AS1 suppression on LUSC progression, as demonstrated by rescue experiments. In addition, the PITPNA‐AS1/miR‐223‐3p/PTN axis accelerated tumor development in vivo.

**Conclusions:**

It is the first time we investigated the potential role and ceRNA regulatory mechanism of PITPNA‐AS1 in LUSC. The data disclosed that PITPNA‐AS1 upregulated PTN through sponging miR‐223‐3p to enhance the onset and progression of LUSC. These findings suggested the ceRNA axis may serve as a promising therapeutic biomarker for LUSC patients.

## Graphical abstract

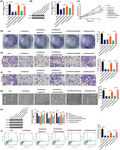

## 1 INTRODUCTION

Lung cancer is one of the leading causes of cancer‐related mortality globally.^1^ Large cell carcinoma, adenocarcinoma, neuroendocrine carcinoma, and squamous cell carcinoma are all types of non‐small cell lung cancer (NSCLC), which accounts for 80–85% of all lung malignancies.^2^ Lung squamous cell carcinoma (LUSC) is a well‐known form of NSCLC with a greater recurrence risk.^3^, ^4^ As a result, identifying molecular biomarkers for LUSC carcinogenesis is paramount.

Long noncoding RNAs (lncRNAs) are RNA molecules with a length of more than 200 nucleotides but no ability to code for proteins.^5^, ^6^ Many studies have shown that lncRNAs are important regulators of gene expression and have a role in the oncogenesis and development of many cancers. For example, via modulating TTN expression, the lncRNA TTN‐AS1 speeds up the carcinogenesis and spread of cutaneous melanoma.^7^ Furthermore, lncRNA ZFAS1 absorbs miR‐892b to regulate LPAR1 and aids carcinogenesis in nasopharyngeal cancer.^8^ FEZF1‐AS1 is a lncRNA that stimulates the Wnt pathway, which promotes gastric cancer carcinogenesis.^9^ The role of lncRNA in LUSC is now the subject of a multitude of research studies. The lncRNA FAM201A, for example, affects LUSC development by altering ABCE1 expression.^10^ Furthermore, the lncRNA HULC promotes LUSC development by upregulating PTPRO.^11^ To speed up LUSC development, the lncRNA NNT‐AS1 targets the miR‐22/FOXM1 axis.^12^ In addition, the lncRNA SNHG1 interacts with TAp63 to modulate ZEB1 expression and exacerbates LUSC metastases.^13^

LncRNA PITPNA antisense RNA 1 (PITPNA‐AS1) has been identified as a new lncRNA that has a role in the progression of a variety of malignancies. In triple‐negative breast cancer, PITPNA‐AS1 has been shown to have an oncogenic role by targeting the miR‐520d‐5p/DDX54 axis.^14^ PITPNA‐AS1 also absorbs miR‐129‐5p to control UNC5B and accelerates papillary thyroid tumorigenesis.^15^ We found that PITPNA‐AS1 has a higher expression level and exacerbates LUSC cell proliferation and migration through interacting with TAF15 to stabilize HMGB3.^16^ In LUSC, the crucial ceRNA regulation mechanism of PITPNA‐AS1 remains to be further explored.

MicroRNAs (miRNAs) are tiny RNA molecules that may control gene expression by interacting with the 3′‐UTR of target genes mRNAs.^17^, ^18^ Several miRNAs have now been demonstrated to have a role in the development of LUSC by acting as tumor promotors or inhibitors. For instance, miR‐30a‐5p inhibits LUSC development by regulating ATG5‐mediated autophagy.^19^ Furthermore, miR‐448 is a prognostic factor that modulates tumor development and metastasis in LUSC via targeting DCLK1.^20^ FGF9 is targeted by MiR‐372‐3p to aid LUSC development and metastasis.^21^ Moreover, miR‐223‐3p has been shown to have a tumor inhibitory effect in LUSC through modulating the miR‐223‐3p/p53 axis.^22^ In LUSC, the relationship between PITPNA‐AS1 and miR‐223‐3p is unknown.

The objective of this research was to look into the biological relevance of PITPNA‐AS1 in LUSC development and the related competing endogenous RNAs (ceRNA) regulatory mechanism. This research might lead to the development of new biomarkers for the treatment of LUSC.

## 2 MATERIALS AND METHODS

### 2.1 Clinical samples

LUSC tissues and adjacent normal tissues were acquired from 74 patients with LUSC. Written informed consents were provided by all the participants. The Ethics Committee of Beijing Tiantan Hospital, Capital Medical University, approved this research (Approval No. KY‐2018‐052‐01). Liquid nitrogen was used to freeze the samples, which were then kept at −80 °C.

### 2.2 Cell culture

LUSC cells (SK‐MES‐1, NCI‐H520, NCI‐H226, NCI‐H2170) and normal BEAS‐2B cells were all acquired from the American Type Culture Collection (ATCC Manassas, VA) and cultured in the Dulbecco's modified Eagle's medium (DMEM, Invitrogen, Carlsbad, CA) and incubated with 95% air and 5% CO_2_ at 37 °C.

### 2.3 RT‐qPCR

Total RNA was extracted from LUSC cells or tissues via TRIzol reagent (Invitrogen), and the cDNA was synthesized using the Primescript RT Reagent (TaKaRa). RT‐qPCR analysis was performed using SYBR®Premix Ex Taq™ Reagent (TaKaRa) through StepOne Plus Real‐Time PCR system (Applied Biosystems). GAPDH or U6 was, respectively, utilized to be lncRNA/mRNA and miRNA internal controls. The fold change in mRNA expression was calculated through the 2^−ΔΔCt^ method.

### 2.4 Cell transfection

The LUSC cells were cultured on 6‐well plates and then transfected with the overexpressed vectors (pcDNA3.1, oe‐PITPNA‐AS1, and oe‐PTN), short hairpin RNA (shRNA) targeting lncRNA PITPNA‐AS1 (sh‐NC, sh‐PITPNA‐AS1), and miR‐223‐3p mimics/inhibitor (miR‐NC mimcs/inhibitor) (GenePharma) through Lipofectamine 2000 (Invitrogen Life Technologies).

### 2.5 CCK‐8 assay

LUSC cell viability assessment was performed through CCK‐8 assay. LUSC cells were cultured on the 96‐well plate, and then CCK‐8 solution was added at 0, 24, 48, and 72 h. Finally, the absorbance at 450 nm wavelength was measured.

### 2.6 Colony formation assay

LUSC cells were seeded onto 6‐well plates. After 2 weeks, the cells were fixed with ethanol and stained with crystal violet (Beyotime). The colonies were then examined under the microscope.

### 2.7 Transwell assay

Transwell chamber ([8.0 μm pore size; EMD Millipore]) with (or without) Matrigel (Becton Dickinson) was applied for the assessment of invasion (or migration) of LUSC cells. The cells in serum‐free medium were placed in the upper chamber, while the lower chamber was filled with medium supplemented with 10% bovine calf serum. Methanol was used to fix the cells after 48 hours, and crystal violet (0.1%) was employed to stain the cells. Finally, the cells that had migrated or invaded were examined under the microscope.

### 2.8 Spheroid formation assay

LUSC cells were cultured into serum‐free low‐adhesion culture plates containing DMEM/F‐12 with N2, EGF (20 ng/mL), and basic‐FGF (20 ng/mL; stem cell medium; PeproTech) to form tumor spheres. The spheres were then observed under the microscope.

### 2.9 Flow cytometry assay (CD44 and CD166)

The CD44 and CD166 antibodies were acquired from Beijing biosynthesis biotechnology CO., LTD. The LUSC cells were maintained in a serum‐free medium. After 1 week, the population of CD44^+^ and CD166^+^‐positive cells was detected through flow cytometry.

### 2.10 Western blot

Proteins were isolated from LUSC cells and tissues using RIPA buffer (Sigma) and then separated using 10% SDS‐PAGE and transferred to PVDF membranes (Millipore). After being blocked with 5% non‐fat milk, the membranes were probed overnight with, anti‐SOX2 (ab97959, Abcam), anti‐OCT4 (ab200834), anti‐Nanog (ab109250), anti‐PTN (ab79411), and anti‐GAPDH (ab8245, Abcam) antibodies at a dilution of 1:1000. Following 3 times washing, the cells were incubated with HRP anti‐rabbit IgG (ab6721, Abcam, 1:2000). Finally, bands were observed with the enhanced chemiluminescence system (ECL, ThermoFisher).

### 2.11 Luciferase reporter assay

The luciferase reporters (PITPNA‐AS1‐WT, PITPNA‐AS1‐MUT, PTN‐MT, and PTN‐MUT) were constructed from Promega. The luciferase reporters were then co‐transfected with miR‐223‐3p mimics (or miR‐NC) in LUSC cells using Lipofectamine 2000. The dual‐luciferase reporter assay system was used to measure luciferase activity 48 h after transfection (Promega).

### 2.12 RNA pull‐down assay

Biotinylated PITPNA‐AS1 probe, PTN probe, and their controls were acquired from GenePharma. These probes, cellular lysates, and M‐280 streptavidin magnetic beads (Invitrogen) were mixed, and miR‐223‐3p expression was detected by RT‐qPCR.

### 2.13 In vivo assay

The nude BALB/c mice (6‐week‐old, 22–25 g) were obtained from the Charles River. The animal experiments were approved by the Ethics Committee of Beijing Tiantan Hospital, Capital Medical University. The LUSC cells were injected subcutaneously into the right lower limbs of mice. Every week, the size of the tumor was measured. After the mice had been euthanized, the volume and weight of tumors were measured.

### 2.14 Statistical analysis

SPSS 22.0 software was applied to perform statistical analysis. The Student's t test (for two groups) or the one‐way ANOVA (more than two groups) was used for statistical comparison. Kaplan–Meier analysis and the log‐rank test were utilized to assess survival curves. The *P*‐value <0.05 was defined as statistically significant. All the data were expressed as the mean ± standard deviation (SD) of 3 replicates.

## 3 RESULTS

### 3.1 LUSC expresses the higher level of LncRNA PITPNA‐AS1

**FIGURE 1:**
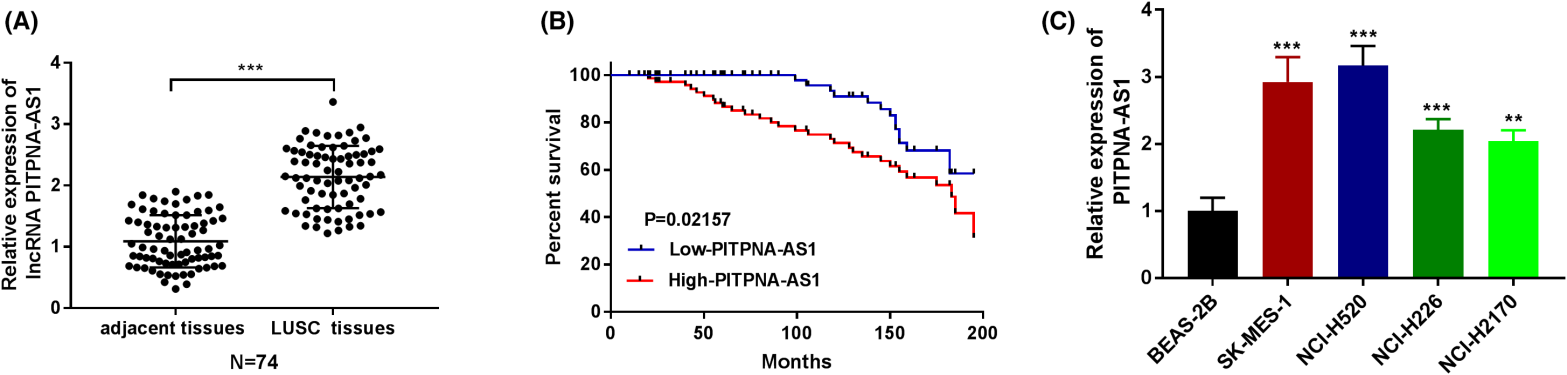
LncRNA PITPNA‐AS1 expression was higher in LUSC. (A) The expression of PITPNA‐AS1 was tested in LUSC tissues and adjacent normal tissues by RT‐qPCR. (B) The relationship between PITPNA‐AS1 expression and the survival rate in LUSC. (C) The expression of PITPNA‐AS1 was verified in LUSC cell lines. **P < 0.01, ***P < 0.001

The 45 LUSC patients' tumor biopsies and associated adjacent normal tissues were collected to investigate the role of PITPNA‐AS1 in LUSC. The findings of RT‐qPCR revealed that PITPNA‐AS1 expression was higher in LUSC tissues (Figure 1A). Furthermore, a higher level of expression was found to be linked with poor prognosis (Figure 1B). PITPNA‐AS1 was upregulated in LUSC cells (SK‐MES‐1, NCI‐H520, NCI‐H226, and NCI‐H2170) compared with normal BEAS‐2B cells (Figure 1C).

### 3.2 PITPNA‐AS1 facilitates cell proliferation, migration, invasion, and stemness in LUSC

**FIGURE 2:**
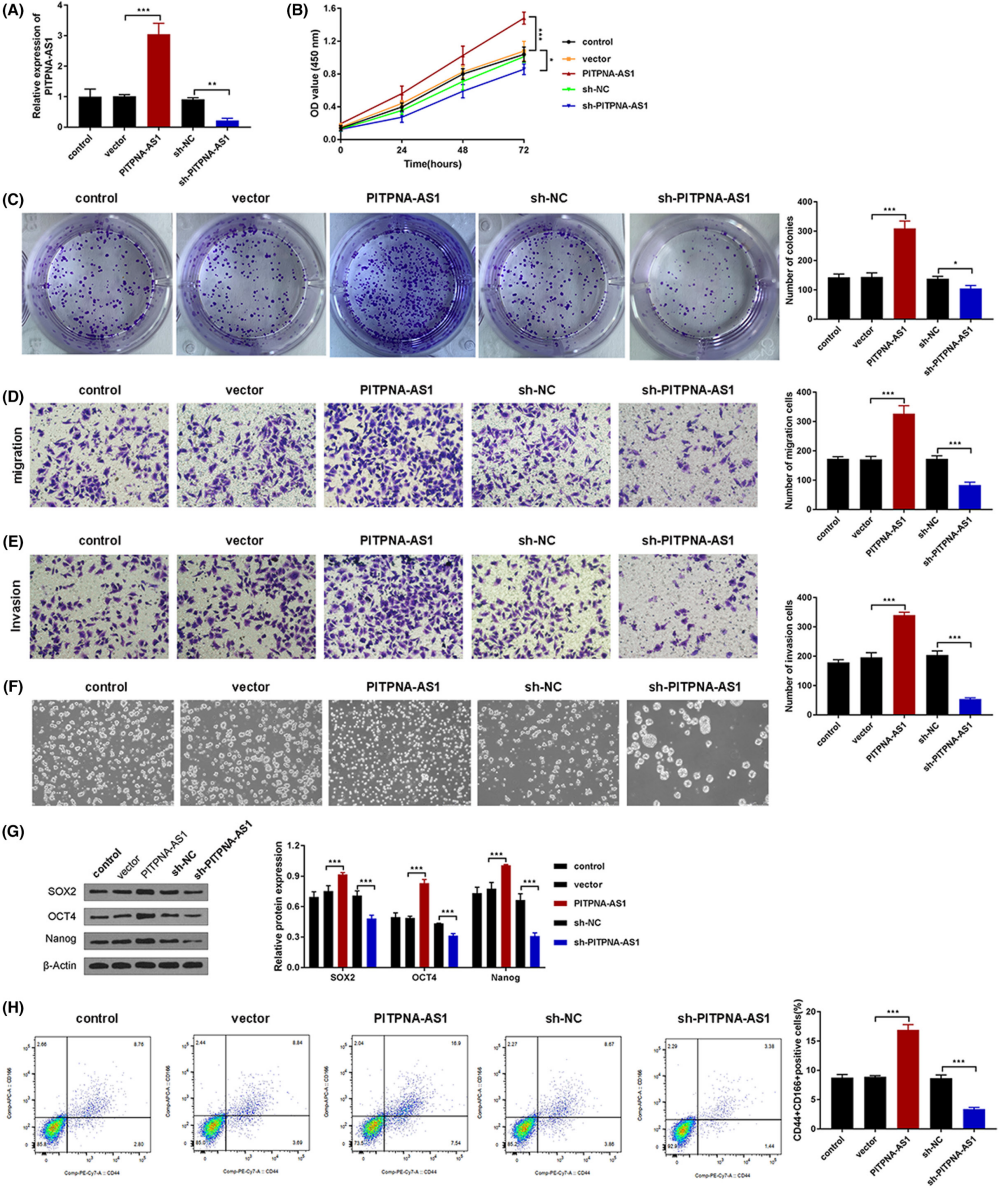
PITPNA‐AS1 contributed to cell proliferation, migration, invasion, and stemness in LUSC. (A) The overexpression and knockdown transfection efficiency was confirmed through RT‐qPCR. (B‐C) The cell proliferation was detected through CCK‐8 and colony formation assay. (D‐E) The migration and invasion abilities were measured through Transwell assay. (F) The stemness of LUSC cells was evaluated through a spheroid formation assay. (G) The levels of SOX2, OCT4, and Nanog were examined through western blot. (H) The CD44^+^ and CD166^+^‐positive cells were detected through flow cytometry. *P < 0.05, **P < 0.01, ***P < 0.001

The shRNA was constructed for knocking down PITPNA‐AS1 (sh‐PITPNA‐AS1) and pcDNA3.1 for upregulating PITPNA‐AS1 (oe‐PITPNA‐AS1), using sh‐NC or vector as a negative control. RT‐qPCR was used to validate the suppression or overexpression efficiency (Figure 2A). It was found that upregulating PITPNA‐AS1 promotes cell proliferation while suppressing PITPNA‐AS1 inhibits cell proliferation (Figure 2B‐C). Furthermore, PITPNA‐AS1 overexpression increased cell migration and invasion, whereas PITPNA‐AS1 inhibition decreased cell migration and invasion (Figure 2D‐E). The findings showed that the formation of tumor spheres was aided by PITPNA‐AS1 overexpression and hindered by PITPNA‐AS1 suppression (Figure 2F). The levels of SOX2, OCT4, and Nanog were increased when PITPNA‐AS1 was overexpressed but reduced when PITPNA‐AS1 was inhibited (Figure 2G). Additionally, following PITPNA‐AS1 upregulation, the number of CD44^+^ and CD166^+^ cells population was increased, whereas after PITPNA‐AS1 suppression decreased CD44^+^ and CD166^+^ cells (Figure 2H). In conclusion, PITPNA‐AS1 increased LUSC cell proliferation, migration, invasion, and stemness.

### 3.3 LncRNA PITPNA‐AS1 absorbs miR‐223‐3p

**FIGURE 3:**
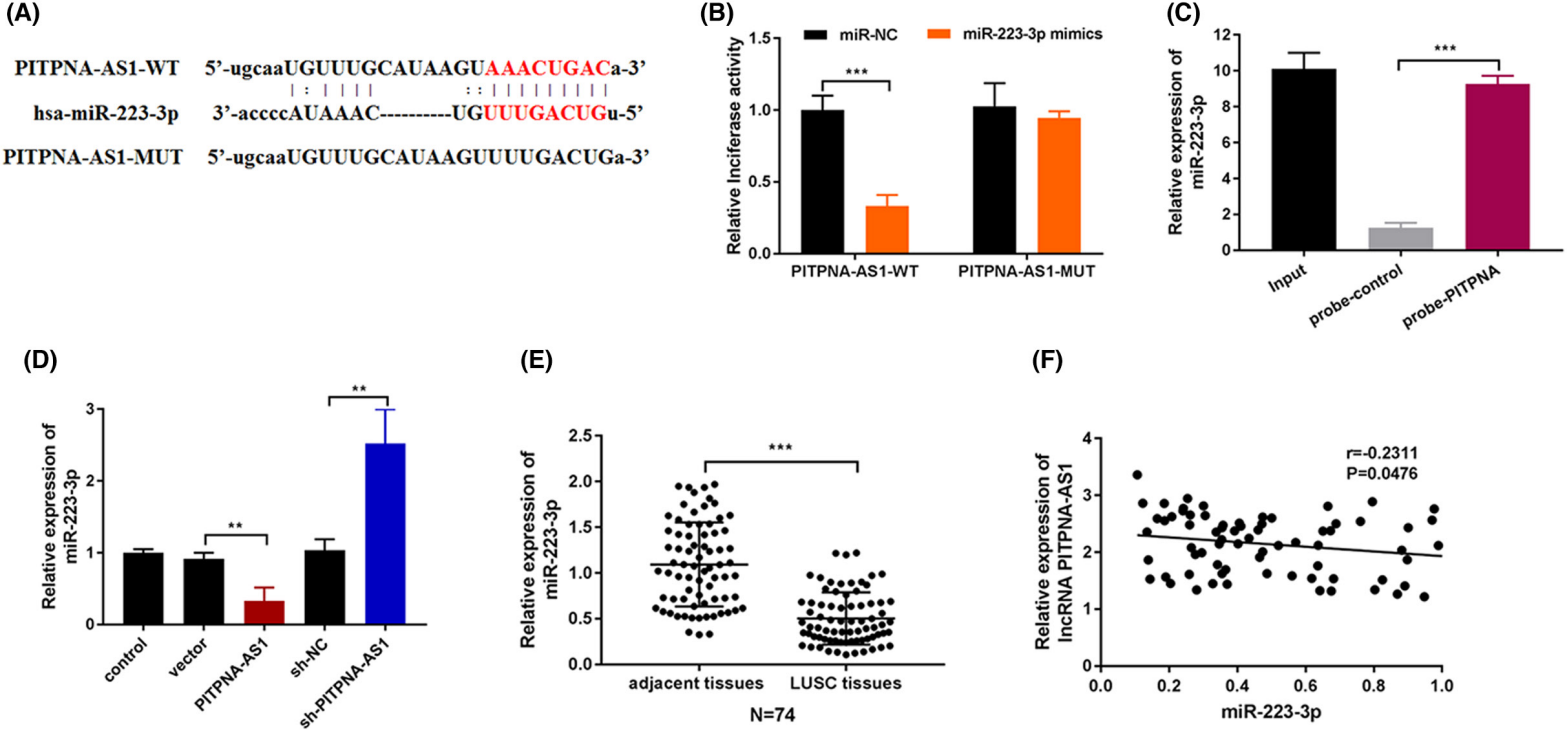
LncRNA PITPNA‐AS1 absorbed miR‐223‐3p. (A) The binding sites between PITPNA‐AS1 and miR‐223‐3p. (B‐C) The binding ability between PITPNA‐AS1 and miR‐223‐3p through luciferase reporter and RNA pull‐down assays. (D) The expression of miR‐223‐3p was tested after overexpressing or suppressing PITPNA‐AS1 via RT‐qPCR. (E) The expression of miR‐223‐3p was assessed in LUSC tissues and adjacent normal tissues through RT‐qPCR. (F) The correlation between PITPNA‐AS1 and miR‐223‐3p was confirmed. **P < 0.01, ***P < 0.001

The regulatory mechanism of PITPNA‐AS1 was further investigated. Figure 3A shows the binding sequences between PITPNA‐AS1 and miR‐223‐3p. It was found that overexpressing miR‐223‐3p reduced the luciferase activity of PITPNA‐AS1‐WT reporters but showed no effect on PITPNA‐AS1‐MUT reporters (Figure 3B). The RNA pull‐down assay also demonstrated that PITPNA‐AS1 absorbed miR‐223‐3p (Figure 3C). After overexpressing (or silencing) PITPNA‐AS1, the miR‐223‐3p expression was downregulated (or upregulated) (Figure 3D). Furthermore, the expression of miR‐223‐3p was decreased in LUSC tissues (Figure 3E). Finally, in LUSC tissues, PITPNA‐AS1 was found to be negatively associated with miR‐223‐3p (Figure 3F). In LUSC, the lncRNA PITPNA‐AS1 absorbed miR‐223‐3p in combination.

### 3.4 MiR‐223‐3p targets PTN

**FIGURE 4:**
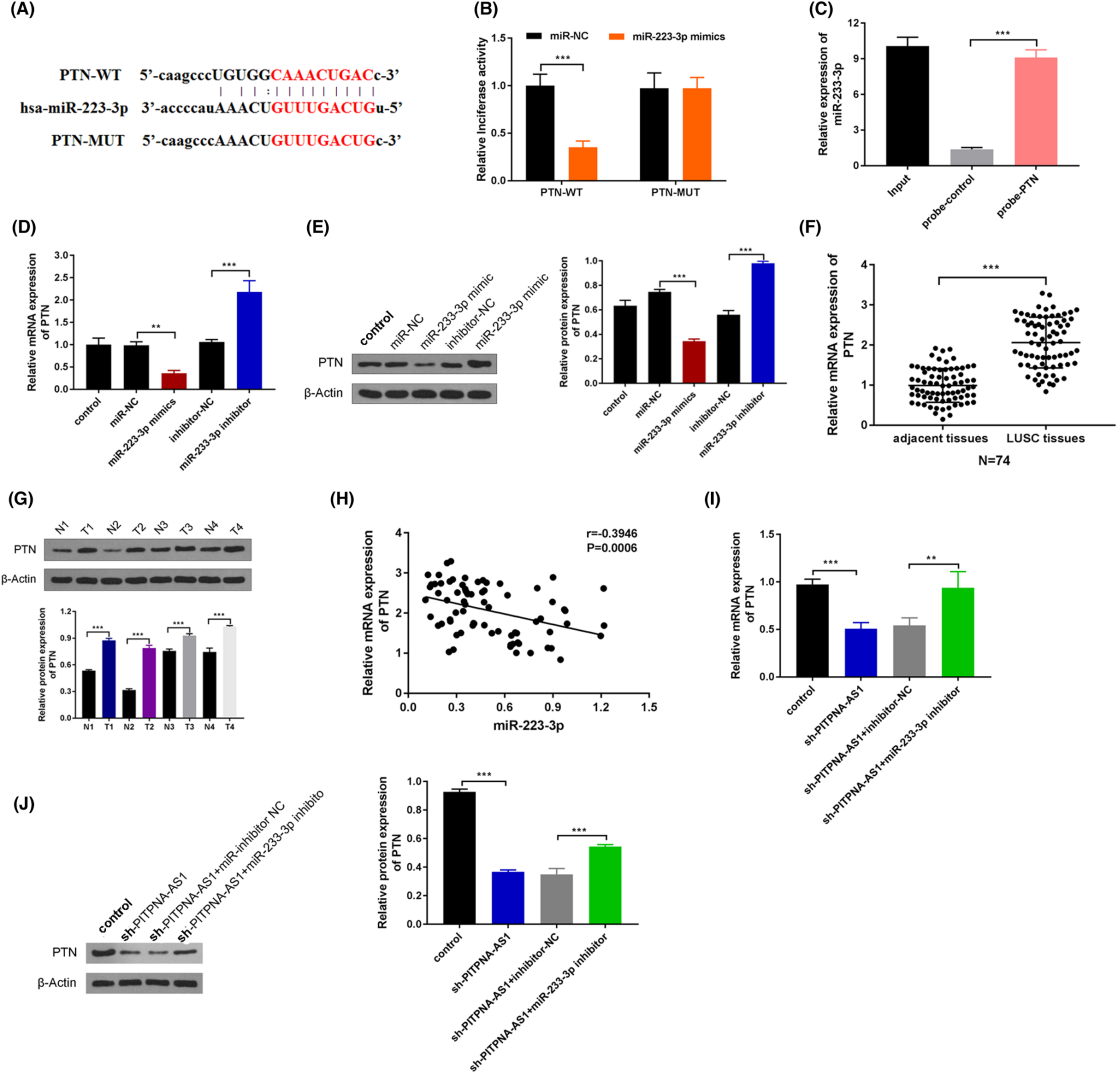
MiR‐223‐3p targeted PTN. (A) The binding sites between miR‐223‐3p and PTN. (B‐C) The binding ability between miR‐223‐3p and PTN through luciferase reporter and RNA pull‐down assays. (D‐E) The mRNA and protein expression of PTN was verified after overexpressing or suppressing miR‐223‐3p. (F) The expression of PTN was assessed in LUSC tissues and adjacent normal tissues by RT‐qPCR. (G) The protein expression of PTN was assessed in LUSC tissues of four patients through western blot. (H) The correlation between miR‐223‐3p and PTN was confirmed. (I‐J) The mRNA and protein expression of PTN were tested via RT‐qPCR and western blot. **P < 0.01, ***P < 0.001

Bioinformatics findings revealed that miR‐223‐3p shared the binding sequences with PTN mRNA 3′‐UTR (Figure 4A). We found that miR‐223‐3p coupled PTN directly as revealed by luciferase reporter and RNA pull‐down assays (Figure 4B‐C). The expressions of both PTN mRNA and protein were also downregulated (or upregulated) when miR‐223‐3p was overexpressed (or inhibited) (Figure 4D‐E). The levels of PTN mRNA and protein were upregulated in LUSC tissues (Figure 4F‐G). In LUSCs, there was also a negative correlation between miR‐223‐3p and PTN expression (Figure 4H). Furthermore, suppressing PITPNA‐AS1 reduced PTN mRNA and protein expression, although this effect could be reversed by inhibiting miR‐223‐3p (Figure 4I‐J). In a nutshell, miR‐223‐3p regulated and targeted PTN in LUSC.

### 3.5 PITPNA‐AS1/miR‐223‐3p/PTN axis regulates cell proliferation, migration, invasion, and stemness in LUSC

**FIGURE 5:**
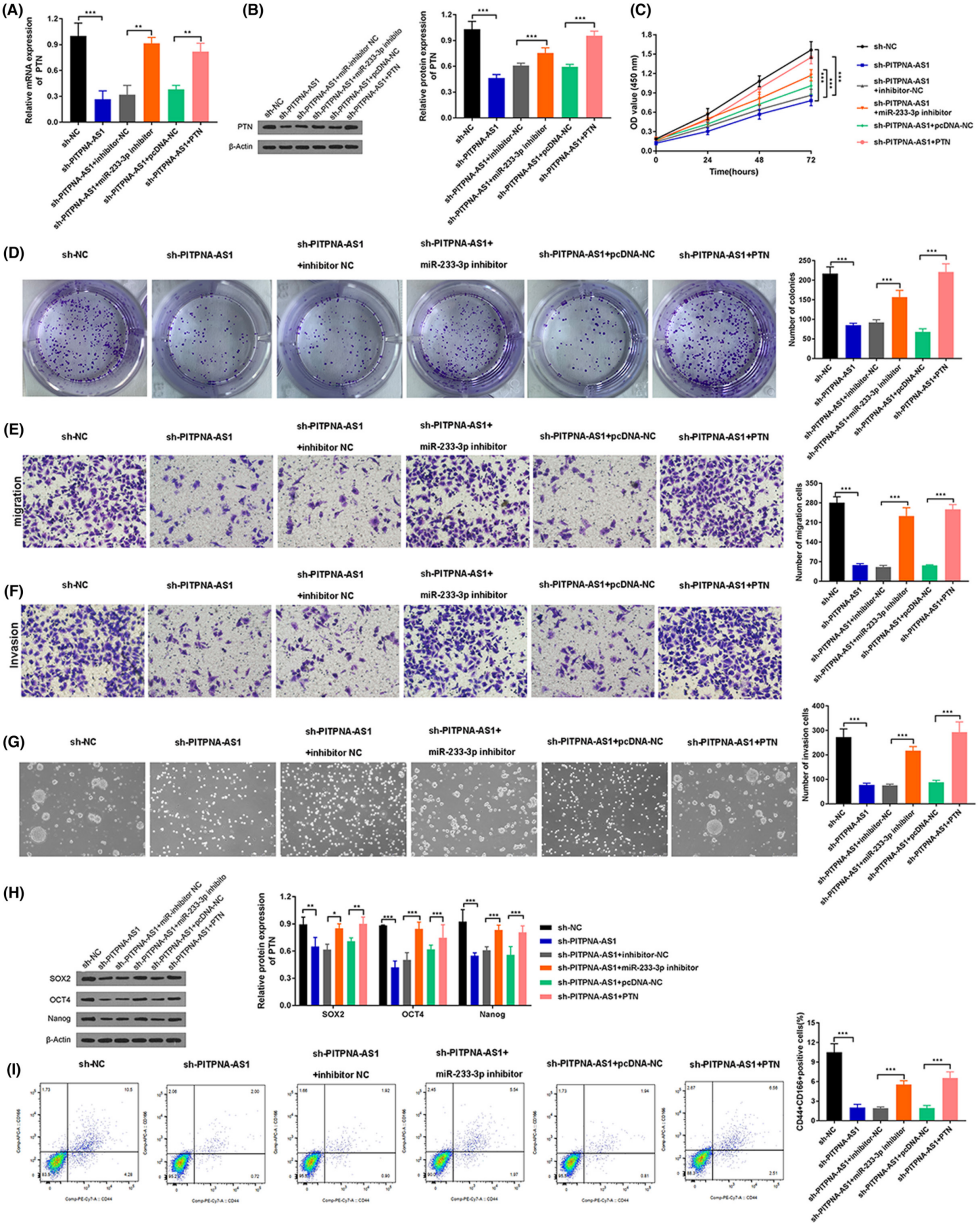
PITPNA‐AS1/miR‐223‐3p/PTN axis regulated cell proliferation, migration, invasion, and stemness in LUSC. Groups were divided into the sh‐NC, sh‐PITPNA‐AS1, sh‐PITPNA‐AS1 + inhibitor NC, sh‐PITPNA‐AS1 + miR‐233‐3p inhibitor, sh‐PITPNA‐AS1 + pcDNA‐NC, sh‐PITPNA‐AS1 + PTN groups. (A‐B) The mRNA and protein expression of PTN were examined through RT‐qPCR and western blot. (C‐D) The cell proliferation was detected through CCK‐8 and colony formation assay. (E‐F) The migration and invasion abilities were measured through Transwell assay. (G) The stemness of LUSC cells was evaluated through a spheroid formation assay. (H) The levels of SOX2, OCT4, and Nanog were examined through western blot. (I) The CD44+ and CD166 + ‐positive cells were detected through flow cytometry. **P < 0.01, ***P < 0.001

Suppressing PITPNA‐AS1 reduced PTN mRNA and protein expression, although this impact may be reversed with miR‐223‐3p suppression or PTN overexpression (Figure 5A‐B). Rescue experiments were used to see whether the PITPNA‐AS1/miR‐223‐3p/PTN axis influences cell proliferation, migration, invasion, and stemness in LUSC. PITPNA‐AS1 knockdown decreased cell proliferation capacity, although this effect may be reversed with a miR‐223‐3p inhibitor or PTN overexpression (Figure 5C‐D). Furthermore, the effects of suppressing PITPNA‐AS1 on cell migration and invasion might be counterbalanced by miR‐223‐3p downregulation or PTN overexpression (Figure 5E‐F). Repression of PITPNA‐AS1 retarded tumor spheres formation, although this impact may be countered by miR‐223‐3p inhibitors or PTN overexpression (Figure 5G). After inhibiting PITPNA‐AS1, the levels of SOX2, OCT4, and Nanog were reduced; however, this effect could be reversed by suppressing miR‐223‐3p or overexpressing PTN (Figure 5H). Furthermore, PITPNA‐AS1 suppression lowered the number of CD44^+^ and CD166^+^ cells, but this effect was reversed by miR‐223‐3p inhibitor or PTN overexpression (Figure 5I). Collectively, the PITPNA‐AS1/miR‐223‐3p/PTN axis increased cell proliferation, migration, invasion, and stemness in LUSC.

### 3.6 PITPNA‐AS1/miR‐223‐3p/PTN axis regulates tumor growth in vivo

**FIGURE 6:**
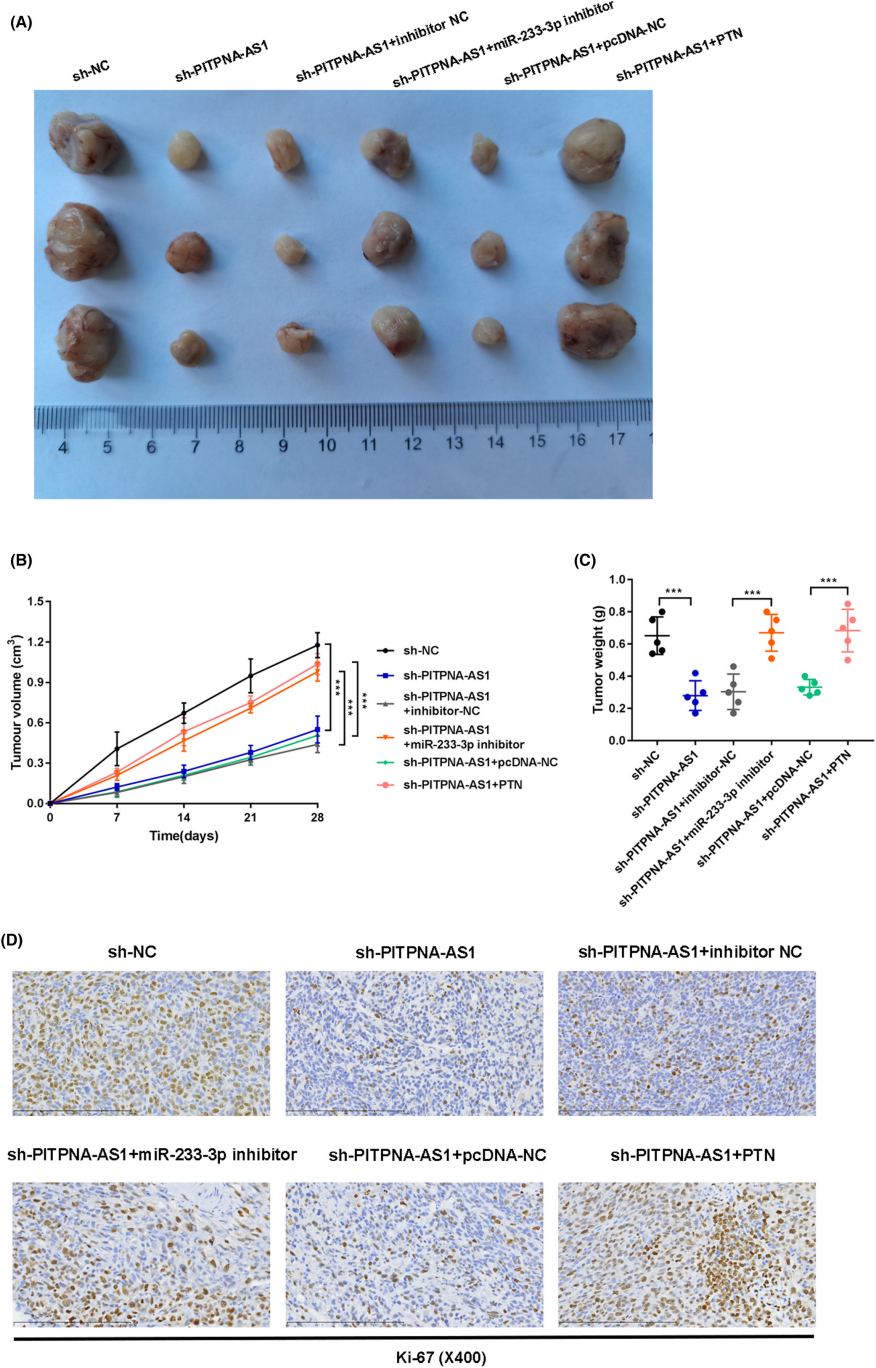
PITPNA‐AS1/miR‐223‐3p/PTN axis regulated tumor growth in vivo. Groups were divided into the sh‐NC, sh‐PITPNA‐AS1, sh‐PITPNA‐AS1 + miR‐inhibitor NC, sh‐PITPNA‐AS1 + miR‐233‐3p inhibitor, sh‐PITPNA‐AS1 + pcDNA‐NC, sh‐PITPNA‐AS1 + PTN groups. (A) The picture of tumors was shown. (B‐C) The volume and weight of tumors were detected. (D) The expression of Ki‐67 was examined through the IHC assay. ***P < 0.001

Finally, we studied the effect of PITPNA‐AS1 on tumor development in vivo. After suppressing PITPNA‐AS1, tumor size, volume, and weight were reduced; however, this effect may be reversed by inhibiting miR‐223‐3p or overexpressing PTN (Figure 6A‐C). Furthermore, the reduced Ki‐67 expression caused by PITPNA‐AS1 knockdown was reversed by inhibition of miR‐223‐3p or overexpression of PTN (Figure 6D).

## 4 DISCUSSION

lncRNAs have received a lot of attention in recent years, and they have played a pivotal role in various malignancies, notably LUSC.^10^, ^11^, ^12^, ^13^, ^14^ PITPNA‐AS1 is a novel lncRNA that has been investigated in LUSC to accelerate tumorigenesis.^16^ However, the ceRNA regulation mechanism mediated by PITPNA‐AS1 in LUSC is unknown. We found that the lncRNA PITPNA‐AS1 expression was higher in LUSC and that this increased expression was associated with a poor prognosis. Additionally, in LUSC, PITPNA‐AS1 facilitated cell proliferation, migration, invasion, and stemness.

LncRNAs share miRNA‐binding regions known as ceRNA, which are used to indirectly regulate mRNAs by absorbing miRNAs.^23^, ^24^ The importance of the lncRNA‐miRNA‐mRNA regulatory network in the development of LUSC has also been discovered.^25^, ^26^ Many ceRNA networks are involved in the LUSC progression. MIR205HG, for example, acts as a ceRNA to accelerate the progression of LUSC by modulating the miR‐299‐3p/MAP3K2 axis.^27^ By regulating YAP1, H3K27ac‐induced LINC00519 absorbs miR‐450b‐5p/miR‐515‐5p to aid LUSC progression.^28^ LINC00355/miR‐466/LYAR ceRNA axis facilitates LUSC development.^29^ The binding sites for PITPNA‐AS1 were found to be shared by miR‐223‐3p. MiR‐223‐3p is involved with a variety of malignancies. For example, miR‐223‐3p activates glycolysis to reduce prostate cancer radiosensitivity by modulating FOXO3a.^30^ Furthermore, miR‐223‐3p binds Arid1a to promote the proliferation and invasion of gastric cancer cells.^31^ MiR‐223‐3p inhibits PRDM1 expression, allowing colon cancer cells to proliferate, invade, and migrate more quickly.^32^ In addition, miR‐223‐3p was revealed to be a tumor inhibitor in LUSC.^22^ In the mechanism exploration, we found that miR‐223‐3p exited lower expression in LUSC, and PITPNA‐AS1 absorbed miR‐223‐3p.

The PTN was shown to be a downstream target gene. According to new findings, PTN is abnormally expressed and acts as a crucial regulator in a variety of malignancies. In osteosarcoma, for example, miR‐627‐3p inhibits cell growth and metastasis by targeting PTN.^33^ T the circ‐LDLRAD3/miR‐137‐3p/PTN axis slows the progression of pancreatic cancer.^34^ Furthermore, the OIP5‐AS1/miR‐137‐3p/PTN axis affects doxorubicin resistance in osteosarcoma.^35^ We further demonstrated that PITPNA‐AS1 negatively controlled PTN expression by absorbing miR‐223‐3p, which is consistent with the preceding results. Furthermore, rescue experiments revealed that inhibiting miR‐223‐3p or overexpressing PTN might alleviate the inhibitory effects of PITPNA‐AS1 silencing on LUSC cell proliferation, migration, invasion, and stemness. In addition, animal studies showed that the PITPNA‐AS1/miR‐223‐3p/PTN axis aided tumor development in vivo.

In conclusion, our findings discovered that PITPNA‐AS1 was upregulated in LUSC and PITPNA‐AS1 accelerated the tumorigenesis of LUSC. Additionally, molecular analysis revealed the PITPNA‐AS1/miR‐223‐3p/PTN regulatory network in LUSC, and PITPNA‐AS1 exerted promotive effects by targeting the miR‐223‐3p/PTN axis in LUSC, providing new insight into the therapy of LUSC.

## AUTHOR CONTRIBUTIONS

YFJ and XJQ analyzed and interpreted data. BHP was a major contributor in writing the article. All authors read and approved the final work.

## CONFLICT OF INTEREST

The authors declared no potential conflicts of interest.

## Data Availability

All the data used to support the findings of this study are included within the article.
